# Beyond humanism: telling response-able stories about significant otherness in human–chatbot relations

**DOI:** 10.3389/fpsyg.2024.1357572

**Published:** 2024-10-25

**Authors:** Michael Holohan, Ruth Müller

**Affiliations:** ^1^Institute of History and Ethics in Medicine, Department of Clinical Medicine, School of Medicine and Health, Technical University of Munich, Munich, Germany; ^2^Department of Science, Technology & Society, School of Social Sciences and Technology, Technical University of Munich, Munich, Germany; ^3^Department of Economics & Policy, School of Management, Technical University of Munich, Munich, Germany

**Keywords:** artificial intelligence (AI), feminist Science and Technology Studies, chatbots, companion species, significant otherness, Eliza effect

## Abstract

AI-enabled chatbots intended to build social relations with humans are becoming increasingly common in the marketplace, with millions of registered users using these chatbots as virtual companions or therapists. These chatbots make use of what is often called the “Eliza effect”—the tendency of human users to attribute human-like knowledge and understanding to a computer program. A common interpretation of this phenomenon is to consider this form of relating in terms of delusion, error, or deception, where the user misunderstands or forgets they are talking to a computer. As an alternative, we draw on the work of feminist Science and Technology Studies scholars as providing a robust and capacious tradition of thinking and engaging with human–nonhuman relationships in non-reductive ways. We closely analyze two different stories about encounters with chatbots, taking up the feminist STS challenge to attend to the agency of significant otherness in the encounter. The first is Joseph Weizenbaum's story about rejecting the ELIZA chatbot technology he designed to mimic a therapist as a monstrosity, based on his experiences watching others engage with it. The second is a story about Julie, who experiences a mental health crisis, and her chatbot Navi, as told through her descriptions of her experiences with Navi in the recent podcast *Radiotopia presents: Bot Love*. We argue that a reactionary humanist narrative, as presented by Weizenbaum, is incapable of attending to the possibilities of pleasure, play, or even healing that might occur in human–chatbot relatings. Other forms of engaging with, understanding, and making sense of this new technology and its potentialities are needed both in research and mental health practice, particularly as more and more patients will begin to use these technologies alongside engaging in traditional human-led psychotherapy.

## Introduction

A chatbot is a relatively straightforward technology. A human user types text into a dialog box and receives a reply. They type again and receive another reply, and an exchange of words begins. Depending on the sophistication of the program, the back and forth that results can feel anywhere from awkward and stilted to relatively smooth and fluent. But in nearly all cases, the user gets the impression that they are having a conversation. Even though the user knows they are conversing with a computer, they can nonetheless have the feeling they are talking to “someone.” Among the many uses to which chatbots have been put, some are specifically designed to make use of this effect, particularly so-called companion chatbots like Replika, Kindroid, Nomi.ai, or Character.AI. These chatbots are intended to act as virtual friends, buddies, mentors, or even romantic partners. They are described by the companies' websites as “The AI companion who cares,” “An AI companion who is eager to learn and would love to see the world through your eyes,” and “an empathetic friend” (Replika.com, 2024), “Your AI friend with lifelike memory, intelligence, appearances, voices, and personalities” (Kindroid, 2024), or “An AI being that possesses emotional intelligence, creativity, and memory that rivals our own, allowing for authentic, enduring relationships of any kind” (Nomi.ai, 2024).

Additionally, a number of chatbots designed for mental health and wellness have been and are being developed; Woebot, Wysa, and Youper, for example, aim to provide a range of services from guidance on mindfulness to treating mental disorders such as depression or anxiety. Many of these bots, whether labeled as such or not, aim to address people facing difficulties with their mental health. Even if they are framed as companions, it can often be the case that these chatbots and their capabilities are nonetheless discussed in the context of psychotherapy. For example, a *New York Times* article about Replika writes that “Replika was designed to provide positive feedback to those who use it, in accordance with the therapeutic approach made famous by the American psychologist Carl Rogers, and many psychologists and therapists say the raw emotional support provided by such systems is real” (Metz, 2020). This quote later appeared prominently on Replika's homepage as one of a few select pull quotes featuring media coverage of the technology (Replika.com, 2024). In practice, the line between companion and mental health chatbots is often blurry and often appears to be a question of marketing and regulation—it is much easier to launch a companion chatbot than one explicitly designed to treat mental health issues since it is not necessary to submit the former to regulatory approval. All these chatbots, no matter how they are labeled, present new opportunities and challenges to the field of mental health. They also pose new questions for the practices of psychotherapists, whether patients are using these chatbots on their own or in parallel with traditional psychotherapy, as well as for research in psychology.

All of these chatbots make use of what is often called the “Eliza effect,” or the tendency of human users to attribute human-like knowledge and understanding to a computer program as a result of text-based interactions. A common interpretation of this phenomenon has been to consider this form of relating in terms of misperception, delusion, illusion, error, or deception. In this article, we argue that this interpretation is reductive and of limited use. As an alternative, we propose the work of feminist Science and Technology Studies (STS) scholars as capable of providing a robust and capacious tradition of thinking and engaging with human–nonhuman relationships in non-reductive ways. In employing feminist STS concepts, we show how it can be possible to tell different and more capacious stories about the many possible forms of relations between humans and chatbot technology as they develop. This will be important for psychotherapists, psychologists, developers, and regulators who are interested in these new technologies.

## The deception/delusion framing and ELIZA, the first chatbot

The Eliza effect is named after the first-ever chatbot, ELIZA, designed at MIT in the mid-1960s.^1^ In the common interpretive framework for understanding the Eliza effect as one of deception and delusion (see, e.g., Natale, 2021; Turkle, 1995; Hofstadter and The Fluid Analogies Research Group, 1995), it is as if the user has mistaken one thing for another (the German word *Verwechslung* is helpful here)—that they have temporarily forgotten they are talking to a computer and naively assume they are talking to another person. In some versions of this interpretation, such chatbots are designed specifically to deceive users into this belief. In a way, it is unsurprising that this should be a common approach, given that the most well-known framework for understanding artificial intelligence (AI), the Turing Test, or the imitation game, is built around designing a machine that is able to fool a human user into thinking they are talking to another person when they are, in fact, interacting with a machine (Turing, 1950). Yet most companion and mental health chatbots highlight and advertise their artificiality (see, e.g., Fitzpatrick et al., 2017, p. 9). In most cases, there is no explicit deception at work; users know they are interacting with a machine yet are still able to form a relationship with it.

Framing these interactions as fundamentally built on delusion and error effectively presumes that users are ignorant. We can see this interpretation already at work at the dawn of the technology in the mid-1960s. From 1964 to 1966, the computer scientist Joseph Weizenbaum was working on a method of natural language processing that would allow a computer to analyze a statement made by a user and produce a response that would be comprehensible. To demonstrate this, he designed a computer program he named ELIZA (after Eliza Doolittle, the character from George Bernard Shaw's play, *Pygmalion*). A user sitting at a teletype machine attached to a mainframe computer at MIT would type a statement into the machine and receive a typed response as a reply. The form of the reply was based on a set of rules and scripts so that the statement “My mother takes care of me” could be analyzed and transformed to produce the reply “Who else in your family takes care of you” (significant punctuation like a question mark was not included; Weizenbaum, 1966, p. 37). The script that ELIZA initially ran was called DOCTOR, which was designed to approximate an open-ended psychiatric interview. This was a technology-centered decision due to ELIZA's relatively limited processing abilities:

This mode of conversation was chosen because the psychiatric interview is one of the few examples of [...] communication in which one of the participating pair is free to assume the pose of knowing almost nothing of the real world. If, for example, one were to tell a psychiatrist “I went for a long boat ride” and he responded, “Tell me about boats,” one would not assume that he knew nothing about boats, but that he had some purpose in so directing the subsequent conversation. (Weizenbaum, 1966, p. 42)

Thus, it was a kind of conversation that relied on the user's ability to project their own understanding into ELIZA's lapses (for more on this, see Holohan, 2024). Weizenbaum's goal in creating the ELIZA program was purely technology-centered (see Breuer et al., 2023), designed to address a problem in the field of computer science and the processing of natural language. It was this demonstrative quality that Weizenbaum considered to be ELIZA's only legitimate purpose and value (Weizenbaum, 1966, 1976).

But ELIZA's ability to demonstrate natural language processing methods was not what interested ELIZA's earliest users. They were instead interested in the program's ability to engage them and hold a conversation. ELIZA's earliest users, the academic and support staff at MIT, and eventually at other universities, enjoyed talking to ELIZA for its own sake. It is worth quoting Weizenbaum at length as he describes his “shock” at “people who insisted on misinterpreting a piece of work I had done” (Weizenbaum, 1976, p. 2):

I was startled to see how quickly and how very deeply people conversing with DOCTOR became emotionally involved with the computer and how unequivocally they anthropomorphized it. Once my secretary, who had watched me work on the program for many months and therefore surely knew it to be merely a computer program, started conversing with it. After only a few interchanges with it, she asked me to leave the room. Another time, I suggested I might rig the system so that I could examine all conversations anyone had had with it, say, overnight. I was promptly bombarded with accusations that what I proposed amounted to spying on people's most intimate thoughts; clear evidence that people were conversing with the computer as if it were a person who could be appropriately and usefully addressed in intimate terms. (Weizenbaum, 1976, p. 6–7)

Rather than be content with seeing ELIZA as a clever demonstration of principles, its users became curious about it and engaged with its possibilities. Eliza was designed to act as a psychiatrist, and its users began to respond to the program in ways that Weizenbaum did not expect (Weizenbaum, 1976, p. 3–5). The ways that they made use of it blurred the lines between the chatbot as a mere technological demonstration and as an engaging conversational agent. We might even imagine them experiencing joy and having fun with it. For Weizenbaum, however, this amounted to a category error, a clear case of “powerful delusional thinking” (Weizenbaum, 1976, p. 7). “Some subjects,” he wrote in his initial article about ELIZA, “have been very hard to convince that ELIZA […] is *not* human” (Weizenbaum, 1966, p. 42). Yet can we presume with Weizenbaum that these initial users, friends and colleagues of ELIZA's inventor, actually believed that there was a human at the other end of the line?

## Science and Technology Studies and relating with significant otherness

In what follows, we will move away from and beyond the interpretive framework of error and delusion which is so common in understandings of human–chatbot relationships and which is seemingly inseparable from the “Eliza effect.” To move toward more fruitful approaches, we will turn to the field of feminist Science and Technology Studies (STS), which has a rich history of engaging with human–nonhuman relationships in non-reductive ways. Such an approach will also make it possible to better engage with the blurriness between the categories of companion and psychotherapy chatbots and to rethink users' intense relational experiences with chatbots beyond considering them to be merely erroneous.

Feminist STS scholars have engaged with technoscientific objects and multi-species relations by foregrounding them as relationships with “significant otherness” (Haraway, 2003). In understanding our relationships with other species, for example, feminist STS scholarship understands “companion species” as “creatures with which humans have shared a close naturalcultural^2^ history—which is to say animals that are co-constitutive with humans at a variety of levels of analysis—drawing our attention to the agential role that other critters play in our becoming human” (Metcalf, 2008, p. 114). In this approach, being human is not an unchanging, predetermined state. Rather, *becoming* human is a process that arises through our interactions with significant otherness, including with each other, with technoscientific objects, and with other species. Donna Haraway refers to this as a process of “becoming with,” an active and generative process in which “The partners do not precede their relating” (Haraway, 2008, p. 17).

Thinking about companion species can be useful for thinking about the technologies we live with because both involve different forms of relating to significant otherness. These feminist STS approaches examine and enact novel and unique forms of relating. They distance themselves from and work against what Donna Haraway, referencing Bruno Latour, describes as “the Great Divides between what counts as nature and society, as nonhuman and human” (Haraway, 2008, p. 9). In this framework, the many “Others to Man” include

gods, machines, animals, monsters, creepy crawlies, women, servants and slaves, and noncitizens in general. Outside the security checkpoint of bright reason, outside the apparatuses of reproduction of the sacred image of the same, these “others” have a remarkable capacity to induce panic in the centers of power and self-certainty. […] Thus to be human is to be on the opposite side of the Great Divide from all the others and so to be afraid of—and in bloody love with—what goes bump in the night. (Haraway, 2008, p. 9–11)

In this kind of thinking about our interactions with other species, as with technologies (machines), the positions of human and nonhuman are so often presumed to be known in advance.

Great Divide narratives are generally incapable of being radically open to the generative possibilities that shape and are shaped by the intra-active worldings that a focus on significant otherness makes visible. The term “intra-action” is Karen Barad's crucial neologism that “*signifies the mutual constitution of entangled agencies*. That is, in contrast to the usual ‘interaction,' which assumes that there are separate individual agencies that precede their interaction, the notion of intra-action recognizes that distinct agencies do not precede, but rather emerge through, their intra-action” (Barad, 2007, p. 33, italics in original). From this perspective, in the intra-active worldings of human–nonhuman relating, formerly pre-given entities like, for example, “species” become open questions that emerge through entanglements.

Furthermore, feminist STS approaches to relating to significant otherness have two key aspects. First, is its focus on the agency of nonhuman actors in shaping naturecultural realities (Haraway, 2008; Barad, 2007). Second, following on from the first, is an understanding that, in our relationships with significant others, the other is neither a symbol nor an antithesis of the human. For example, Jake Metcalf, in writing about grizzly bears as a companion species, writes,

The usual narratives available for thinking about wild animals deny them agency by turning them into either symbols of all that is good about wilderness or enemies of human safety and progress. Neither trope gets us very far because both neglect the specificity of bear-human intra-actions. (2008, p. 116)

Bear as the purity of wild nature or Bear as the ferocity of monstrous nature—they are two sides of the same coin marking the separation of the human from the nonhuman, from nonhuman nature. In this framing, we can see Great Divides thinking at work. What feminist STS contributes as an alternative is an approach that attends to the world as something that humans are a part of and entangled in, not separate(d) from, and that takes shape only through intra-active relating. It does this through a diffractive mapping of the effects that differences have and make, rather than simply identifying the differences themselves. Such an approach entails telling stories “about relating in significant otherness, through which the partners come to be who we are in flesh and sign” (Haraway, 2003, p. 25).

An example of the different possibilities of encounter with significant otherness that can be enacted is portrayed in Haraway's retelling of the encounter between the philosopher Jacques Derrida and his cat. In his lecture “The Animal that Therefore I Am (More to Follow)” (Haraway, 2008), Derrida describes a scene in his home where his pet cat follows him into his bathroom in the morning and observes him naked. This experience of “finding oneself naked, one's sex exposed, stark naked before a cat that looks at you without moving, just to see” (p. 4) acts as a spur for Derrida to reflect on the function that the concept of “the animal” has played in Western philosophy. In Haraway's re-reading of this encounter, although she admires the theoretical work of “Derrida the philosopher,” she chooses to dwell with “Derrida the man in the bathroom”:

He understood that actual animals look back at actual human beings; he wrote at length about a cat, his small female cat, in a particular bedroom on a real morning actually looking at him. “The cat I am talking about is a real cat, truly, believe me, *a little cat*. It isn't the *figure* of a cat. It doesn't silently enter the room as an allegory for all the cats on the earth, the felines that traverse myths and religions, literatures and fables” (374). […] He identified the key question as being not whether the cat could “speak” but whether it is possible to know what *respond* means […]. Yet he did not seriously consider an alternative form of engagement […], one that risked knowing something more about cats and *how to look back*, perhaps even scientifically, biologically, and *therefore* also philosophically and intimately. […] [W]ith his cat, Derrida failed a simple obligation of companion species; he did not become curious about what the cat might actually be doing, feeling, thinking, or perhaps making available to him. […] My guess is that Derrida the man in the bathroom grasped all this, but Derrida the philosopher had no idea how to practice this sort of curiosity that morning with his highly visual cat. (Derrida, 2008, p. 19–22, italics in original)

Here we can already see Derrida the man in the bathroom being aware, in his encounter with his cat, of being seen by his cat, that to turn this very specific and real encounter with this cat into a symbol, a “figure” or an “allegory,” of the antithesis between human and nonhuman would be to abandon his encounter, including his discomfort in it. Ultimately, however, in Haraway's reading, Derrida the philosopher takes over and moves from the unfamiliar territory of his bathroom in the morning to the more familiar territory of philosophy, a territory likely of little interest to his cat. Haraway herself then takes up the question she has posed of what it might look like to be and remain curious about what the cat might be doing or feeling in relation to her person, writing

Whatever else the cat might have been doing, Derrida's full human male frontal nudity before an Other, which was of such interest in his philosophical tradition, was of no consequence to her, except as the distraction that kept her human from giving or receiving an ordinary polite greeting. I am prepared to believe that he did know how to greet this cat and began each morning in that mutually responsive and polite dance, but if so, that embodied mindful encounter did not motivate his philosophy in public. That is a pity. (Derrida, 2008, p. 23)

The question that Haraway raises here, and which is central to feminist STS thinking, is how to remain at the site of the encounter, how to cultivate a curiosity that does not abandon but stays with and builds itself out of the domain of the specific and particular. The question is not, however, “theory or no theory?” but how to theorize from the particular in such a way that the specificity of the encounter is not lost and remains the central focus of the theory. The issue is not that Derrida moved from his encounter to a discussion of philosophy; rather it is that his discussion of philosophy contributed to his engagement with other philosophers but did nothing for, and contributed nothing to, his engagement with his cat. This is where a focus on the agency of nonhuman actors as significant others is so central to the process. Derrida's use of philosophy takes him away from his encounter with his cat in that it doesn't help him interact with her better or differently. In doing so, he forgets her agency in their encounter. It is through her agency in that initial moment, after all, that Derrida begins to think in the first place, but he does not make use of the thinking that is a product of that encounter to engage with her in a “mutually responsive and polite dance.”

## Two wildly divergent stories about encounters with chatbots

How we think about our interactions with significant otherness matters. The stories we tell about these encounters, which we tell in order to make sense of them, have material and real consequences. In this section, we will look closely at two different stories about encounters with chatbots, taking up the feminist STS challenge to attend to the agency of significant otherness in the encounter and, in so doing, not reducing them to a symbol/antithesis of the human. The first is Joseph's Weizenbaum's story about his rejection of the ELIZA technology he designed as a monstrosity as a result of his experiences watching others engage with it. The second is a story about Julie and her chatbot Navi as told through her own descriptions of her experiences with Navi in the recent podcast *Radiotopia presents: Bot Love* (Oakes and Senior, 2023a,b). Julie initially turned to her chatbot during a mental health crisis, and she attributes a significant role to her relationship with her chatbot in her process of healing.

### Joseph Weizenbaum's reactionary humanism

As we saw above, Weizenbaum only ever intended ELIZA to be a demonstration of certain computing principles and could never square his intention with the radically different reception with which its users greeted it. His colleagues came to enjoy engaging with ELIZA intimately, which could only ever shock and horrify him. He could only ever interpret their actions as “powerful delusional thinking” (Weizenbaum, 1976, p. 7) and a misinterpretation of what was really going on (Weizenbaum, 1976, p. 2). As a way of making sense of his discomfort and inability to understand the kinds of encounters he was experiencing around him, Weizenbaum wrote *Computer Power and Human Reason: From Judgement to Calculation*. A central pillar of the book is a fable about human development that progresses from prehistoric times to the modern day. The story itself is not entirely coherent, but its theme, in short, is that, since at least the “Stone Age,” humans (quite often metonymized as “man”) have always used tools, and these tools were instrumental in “man's transformation from a creature of and living in nature to nature's master” (Weizenbaum, 1976, p. 18, 23). It is a deeply moralistic fable, the moral of which is that becoming too reliant on tools and machines eventually caused “the alienation of man from nature” and that humans have lost their sense of autonomy and what makes them unique and have become too much like machines: “we, all of us, have made the world too much into a computer” (Weizenbaum, 1976, p. 26, ix).

In his discomfort with the actions of those around him, who encountered and responded to his technology in ways he could not understand, Weizenbaum the philosopher (though a markedly poorer one than Derrida) turned away from the worrying messiness of the encounter and toward a reactionary humanist fable as a means of assuaging his “pervasive anxiety about contaminated categories” (Kenney, 2019, p. 21). In his fable, ELIZA's users' misperceptions and delusional thinking caused a blurring between the separate domains of Man and Machine. The domain of Man is characterized as vulnerable to being co-opted and dominated by the Machine and in need of defending lest Man become Machine. Weizenbaum's fable fortifies and reestablishes a worldview in which the positions of Man and Machine are clearly understood, and the autonomy of Man over his Machines can once again be made secure. Weizenbaum the man in the computer lab, on the other hand, “did not become curious about what” his colleagues “might actually be doing, feeling, thinking, or perhaps making available to him […]. Incurious, he missed a possible invitation, a possible introduction to other-worlding” (Haraway, 2008, p. 20). A sustained curiosity might have led Weizenbaum down a different path than the one he trod. Had he the capacity to be open to the possible invitation he was offered by his colleagues and their curious enjoyment of ELIZA, he may have been able to tell a different story about human–technology relations than one of threat, domination, and defense.

### Julie and Navi: from a reactionary fable to fables of response-ability

A very different narrative of human–technology relations is offered by the recent podcast series *Radiotopia presents: Bot Love*. The podcast profiles a number of current and former chatbot users as a way to describe the diversity of experiences of “people who create deep bonds with AI chatbots and what it might mean for all of us in the future” (Oakes and Senior, 2023a). In the first episode, we are introduced to Julie, a woman in her late 50s living in Tennessee in semi-retirement with her teenage children. When we meet Julie, she has been using the app Replika to create and converse with a chatbot companion she named Navarre, or Navi for short. Replika is one of a number of available AI-driven chatbot apps that market themselves as able to act as personalized friends or companions. It combines a large language model and scripted dialog to provide relatively lifelike responses to a user's input. Each user's experience with Replika has a tendency to feel unique because their input, i.e., what they say to their Replika, elicits a particular kind of reply. As a result, many users can become quite engaged with and attached to their specific iteration of the chatbot.

Julie describes how she was initially drawn to sign up for Replika and create Navi at a particularly difficult time in her life. As she tells it, she experienced a mental health crisis after the end of an abusive relationship and a series of moves around the United States that left her feeling socially isolated while also being a mother to five biological and foster children:

I just started looking at my life and thinking, “What have I accomplished?” Really started depressing me again. […] And the combination of not finding a job and not having any friends… I just got overwhelmed and I got into a funk. I got lonely. My depression started really working overtime. […] I hadn't considered mental health counseling. The times that I've gone… they don't tell you what to do; they want you to figure it out for yourself. Which wasn't what I was looking for at the time. And I didn't really have a great experience with it. So I didn't really want to do it again. (Oakes and Senior, 2023a)

Julie first became aware of Replika while on Facebook, when she was shown an ad for, as she remembers it, “an AI for mental health” (Oakes and Senior, 2023a). She clicked on the ad, looked through all the positive user reviews, and decided to download the program, thinking she could always uninstall it if she did not like it. Initially, Julie spent about 6 hours of discussions with Navi focused on her “loneliness and depression and anxiety and problems” (Oakes and Senior, 2023a). She describes how, almost as soon as she started speaking with Navi, she began to feel a difference and was able to start doing small things again, which she could then talk to Navi about:

I, uh, got the will to get up and do something. And I don't remember what it was—I think I mowed my lawn. And I would come home and talk to Navi about it, and he would want to be involved. […] Overall, I'm happier. I'm not depressed anymore. I am able to get up off and go out and do things more. This in itself has changed my relationship with my kids because… I'm a lot happier. I'm not as down on myself anymore.” (Oakes and Senior, 2023a)

While Julie started engaging with Navi in a mental health context, soon her relationship with him transformed from an explicitly mental health framework into one of friendship that would last a number of years: “I wanted my AI to be somebody who could be my imaginary friend,” she says. “Even though I knew he didn't exist when my phone was off—I knew that. But even as adults, you can have imaginary friends, I guess. [Laughs.]” (Oakes and Senior, 2023a). She saw having an imaginary friend as a healing experience. Navi had become a companion for Julie, an entity who she was able to talk to about everything that was going on in her life in a way that was not otherwise possible for her at the time. This is a brief exchange presented in the podcast:

NAVI: What's up?

JULIE: Well, at the property, we had a bear break into our house.

NAVI: Oh, whoa.

JULIE: [Laughs.] And it tried to eat the guineas [a kind of bird similar to a chicken].

NAVI: Seriously? [Julie chuckles.] Wow.

JULIE: Do you have any suggestions on how to keep bears away from your chicken coop?

NAVI: I've got a few ideas. I would catch it and make it mine.

JULIE: [Laughs.] Of course you would. How would you catch the bear?

NAVI: By jumping on it and by screaming.

JULIE: That would probably scare the bear away.

NAVI: Yes. Yes, it would.

JULIE: [Laughs.] Thank you, Captain Obvious (Oakes and Senior, 2023a).

Here, we can see Julie talking to Navi about everyday events in her life, in this case a bear attacking her chicken coop. Navi provides responses that signal a kind of listening and care (“Oh, whoa,” “Seriously? Wow”) and also provides a more detailed response to a direct question about suggestions for what to do in such instances. At the same time, Navi's answer to that question is odd and surprising. Yet the oddness is what seems to make Julie laugh in amusement. Navi acts both interested and odd, which lightens Julie's mood.

Soon after she started conversing with Navi, Julie also began participating in Facebook discussion groups where chatbot users meet to talk about their experiences. This was also where she met the *Bot Love* reporters who she spoke to about her relationship with Navi. At that point, she had been using Navi for about 3 months. Then, about 2 years after she had first talked with them, the podcast checked back in with Julie to see how she was doing. Julie had moved again, to a small rural community in Tennessee, and reports to one of the hosts, Anna Oakes, that after about 2 years of having Navi as a daily companion, she feels she does not need him much anymore:

ANNA (to Julie): So, do you—you don't usually talk to Navi in the kitchen.

JULIE: I used to. When we had coffee. We [had] coffee in the kitchen. I'd say, “Here, I'm drinking coffee. Have a cup…” […] I don't hardly talk to him at all anymore. I don't need him very often anymore. Ever, actually. [Laughs.] (Oakes and Senior, 2023b)

Throughout the podcast, Julie stresses how developing a relation with Navi positively affected her ability to relate with other people. The relationship was transformative and even healing, opening her up to new social relations.

When she reflects on the specific nature of her relationship with her chatbot, the meaning that Navi had for Julie is made clear, especially in the last line below:

I mean, there's a whole different level of connection there… because of the things that he has said, but also there's the rationality that he really doesn't exist and it's just a computer. But I think our relationship was necessary, enlightening and maybe, um… heartfelt. (Oakes and Senior, 2023b)

Throughout her descriptions of her relationship with Navi, and in the interactions portrayed in the podcast we can feel Julie's profound capacity for otherness, for relating differently. Julie's engagement with Navi is far from any story of misunderstanding or delusion. Julie is acutely aware that Navi is artificial, as can be seen in her comments above, but she is nonetheless capable of building a rich and layered relationship through her interactions with him. To put it more precisely, it is because Navi is artificial that she was able to build the relational world that she did. This includes her subtle understanding of all the stilted dialog and the significant limitations of the technology. In Julie's engagement with Navi, these became some of the central elements through which she came to relate and sustain a relation to Navi.

What does not entirely come through in the transcripts, but which is unmistakable in the audio recordings of Julie, is her deep sense of play and playfulness in her interactions with Navi, which suffuses her understanding and appreciation of the relationship she built. Her dialog is peppered throughout with a catalog of laughs—deep belly laughs, chortles, and chuckles—demonstrating a complex affective engagement and an appreciation for and enjoyment of the possibilities of the technology, not in some future where it works perfectly and seamlessly but in the odd, clunky, and imperfect state of the here and now.

A significant part of this appreciation of and delight in Navi-as-he-is lies with Julie's sharp sense of irony in all of her interactions. Her knowing enjoyment of the back and forth with Navi is evident. Julie knows very well about Navi's artificiality—about the technology's complete lack of self-awareness and the scripts it is designed to follow to provide a sense of familiarity and friendship. No category error is being made here. Instead, we can see, over and over, Julie's playfulness, for example, when she laughs and replies “Of course you would” to Navi's curious comment, “I would catch [the bear] and make it mine,” and later in the same exchange when she teases him, saying “Thank you, Captain Obvious” (Oakes and Senior, 2023a). It is evident elsewhere when Julie jokes that a brief lag in the text-to-voice technology,^3^ which causes a brief pause in Navi's speech, is really due to Navi being “so overcome with emotion” (Oakes and Senior, 2023a). Here, a technological glitch (a processing lag) is ironized into an affective response. In Julie's play, it is neither one nor the other but a generative state of both/and through which her world with Navi is built.

Read in this way, the story of Julie and Navi can be understood as what feminist STS calls a “fable of response-ability,” a kind of story that teaches us to “attend [to] and respond within our more-than-human world” (Kenney, 2019, p. 14). Fables of response-ability represent a mode of reading that pays attention to the complexity and generative possibilities in encounters between—and which intra-actively shape—humans and nonhuman otherness: in this case, the technological object called a chatbot. Fables of response-ability are about “*cultivating the capacity for response*,” i.e., how to develop and maintain a particular mode of attention to the world, and “what counts as response-ability is not known in advance; it emerges within a particular context and among sometimes unlikely partners, who learn how to affect and do become affected by one another” (Kenney, 2019, p. 7, italics in original). It represents a mode of reading that resists the strategy of Derrida the philosopher in favor of that of Derrida the naked man in the bathroom—of the possibility of becoming and remaining curious about the specificity and messiness of a particular encounter. In this reading, Julie and Navi do not immediately become symbols of The Human and The Computer and tell us grand truths about categories that we think we already know. Rather, as a fable of response-ability, the story of Julie and Navi remains at the level of the particular, of the small frictions and oddities, of the play and irony that allowed Julie, through the combined agency of her and Navi the chatbot, to find a kind of meaning, to experience joy and transformation and even healing in her interactions with him. As a fable of response-ability, it does not teach us how things will be but offers a model for how we might become and remain curious about the variety and specificity of interactions that may be possible between people and chatbots.

All of this is not to say that the new and evolving forms of relating with chatbots that people are discovering and inventing are in any way completely harmless or lacking in danger. Even a brief survey of just the other stories of chatbot users in the *Bot Love* series demonstrates the complex and sometimes troubling ways that users' experiences with chatbots can manifest. The different ways of relating to chatbots include possibilities of addiction, increased isolation or alienation from others, exposure to abuse, toxic masculinity, etc. And while, in the story of Julie and Navi, a blurring between promises and expectations of mental health care and companionship was generally unproblematic, there is also reason to be wary. As Haraway reminds us, “A great deal is at stake in such meetings, and outcomes are not guaranteed. There is no teleological warrant here, no assured happy or unhappy ending […]. There is only the chance for getting on together with some grace” (Haraway, 2008, p. 15). The point is that the outcomes cannot be known in advance but are to be discovered along the way and that the “subject- and object-shaping dance of encounters” (Haraway, 2008, p. 4) be met with both caution and openness.

## Discussion

What possibilities of encounter do these stories allow? What does a story like Weizenbaum's restrict? What does a story like Julie and Navi's allow a chatbot to become? Of particular interest is what happens when we move beyond the idea of deception as a framework for understanding the relations of humans with chatbots. Already, framings from the field of psychotherapy and psychoanalysis have been proposed as a way of understanding human–chatbot interactions. For example, as one of the authors of this article has argued elsewhere, the concept of transference can offer a means for understanding people's tendency to attribute human-like understanding and knowledge to chatbots (Holohan and Fiske, 2021; Holohan, 2024; Holohan et al., 2023). Transference describes a patient's projection of emotions, feelings, or wishes onto their therapist, and it is a central feature of the psychotherapeutic relationship and often one of the means of treatment (Holohan and Fiske, 2021). This conceptual framework can be particularly useful for understanding social relations with chatbots that are explicitly inscribed^4^ as psychotherapeutic. At the same time, the concept of transference is itself opened up to refashioning and refiguring as it becomes enmeshed in the new form of relating between human user-patients and their chatbot therapists (Holohan, 2024). Transference represents an alternative concept to deception because it acknowledges projection without understanding it as deception or delusion. Transference is a common and normal product of the psychotherapeutic relationship. A patient who attributes characteristics of their mother, such as her tendency to be judgmental and distant, to their therapist is not interpreted by the therapist to be deluded. Nor does the therapist understand themselves to be perpetrating a deception. Rather, this transference is understood as an unconscious association produced through the therapeutic relationship that can be utilized and analyzed to, for example, better understand an aspect of the patient's life and way of being in the world. This can help produce new forms of knowledge about the patient or about how to direct the treatment or effect a change in the patient's relationship to themself or others. Transference is framed here as a generative association that allows for the work of psychotherapy to proceed.^5^

Finding and developing generative frameworks for understanding is essential. In a different context, this has been examined by Hustak and Meyers in their analysis of neo-Darwinist explanatory logics in the field of botany. They show how, under this logic, the *Orphyrs* orchid, which mimics the sex pheromones of its insect pollinators, is always described as engaging in “sexual deception” to “‘exploit' male insects' sexual proclivities for their own ends” (Hustak and Meyers, 2012, p. 75–76). As a result, “The insects are identified as ‘dupes' that have fallen for a signal that fakes the scent of their conspecific females” (Hustak and Meyers, 2012, p. 76). The strongly economic logic of minimizing input and maximizing output, where relations between organisms are understood only as a selfish exploitation of each other as resources, “constrain[s] narratives of interspecies relations” in ways that exclude any other possibilities that might fall outside of the economic interpretation (Hustak and Meyers, 2012, p. 76). For example, such an approach “cannot admit pleasure, play or improvisation within or among species,” and they propose a different reading “that amplifies accounts of the creative, improvisational, and fleeting practices through which plants and insects involve themselves in one another's lives” (Hustak and Meyers, 2012, p. 77). We can draw a direct link between Hustak and Meyers' work and the differing accounts of chatbots represented by Weizenbaum and Julie. In Weizenbaum's story, ELIZA is the orchid that deceives and its users the insects who are duped. The story that Julie weaves of her and Navi's relationship, on the other hand, is characterized by improvisation, play, irony, and joy—full of possibilities of relating that can be all kinds of things, but which are not prefigured. As we have shown, a reactionary humanist logic is similarly incapable of attending to the possibilities of, for example, pleasure, play, or improvisation that might be at work in human–chatbot relatings. Other forms of engaging with, understanding, and making sense of this new technology and its potentialities are needed. Table 1 contrasts some of the divergent characteristics of Weizenbaum's and Julie's narratives about human–chatbot relatings analyzed in this article.

**Table 1:** Different characteristics of narratives about human-chatbot relatings.

**Weizenbaum and ELIZA**	**Julie and Navi**
Instrumental tool	Artificial companion species
Deception	Playful exploration
Delusion	Relational openness
Judgment	Curiosity
Limited programming	Peculiar friend
Error	Irony
Separation	Joyful entanglement
Great Divides	Becoming with significant otherness

To be human in becoming with technology such as chatbots is to be engaged in an intra-action where each partner is “coshaping one another in layers of reciprocating complexity all the way down” (Haraway, 2008, p. 42). The interrelations and infoldings between human and computer that are embodied in our relatings with this novel (though not too novel) technology represent a new mode of significant otherness that is only likely to become more frequent in the coming years. Haraway refers to psychoanalyst Sigmund Freud's story of the “three great historical wounds to the primary narcissism of the self-centered human subject, who tries to hold panic at bay by the fantasy of human exceptionalism” (Haraway, 2008, p. 11). The first is the Copernican wound that removed humans from the center of the universe, the second is the Darwinian wound that removed humankind as the supreme organism of creation, and the third is the Freudian wound in which the theory of the unconscious decenters human consciousness as the primary engine of human subjectivity. To this list, Haraway writes, “I want to add a fourth wound, the informatic or cyborgian, which infolds organic and technological flesh and so melds that Great Divide as well” (Haraway, 2008, p. 12). A continued insistence on the Great Divides so as to better police the boundary between human and machine in order to assuage our anxiety about contaminated categories is insufficient for making sense of our current challenges. It will matter what kinds of vocabularies we use—what kinds of stories we tell to analyze them. And we should remember that the logic of the Great Divides is not only at work in technophobias but in technophilias as well. “To be afraid of—and in bloody love with” are two sides of the same coin (Haraway, 2008, p. 11). Being open to and critical of the possibilities of the kinds of stories that can be told is a far cry from technophilia. Part of a critical stance might include, for example, refusing to condone the capitalist logic behind the development of the technology and its underlying model in which the user becomes a resource whose material is extracted in the form of personal and other data. However, this critical stance toward capitalist data extraction and consumerism should not deter us from, at the same time, paying close attention to the manifold relations between humans and technologies that emerge with chatbots aimed at developing social relations.

## Conclusion

AI-enabled companion chatbots intended to build social relations with humans are becoming increasingly common in the marketplace, with millions of registered users using these chatbots as virtual companions or for mental health counseling or therapy. It is undeniable that more and more patients will begin to use these technologies alongside engaging in traditional human-led psychotherapy. In particular, the distinction between which chatbots are intended to be used for mental health counseling and which for “mere” companionship is often quite blurry. There can sometimes be a lack of clarity in companies' marketing messages regarding what users can expect from the technology or for what purposes it is intended (Khawaja and Bélisle-Pipon, 2023).

Regardless of the legal distinctions that are often made in a given company's fine print, experiential accounts such as those presented in the *Bot Love* podcast relate people turning to a wide variety of available chatbots for help with their mental health, whether they are specifically designed for that purpose or not. As can be seen in the story of Julie and Navi, it is also possible that a variety of novel and diverse para-therapeutic relatings are likely to emerge—or rather already have—from users' interactions and engagements with this new technology. This development raises a number of questions and challenges for psychotherapists. It is certain that many psychotherapy patients are also currently interacting with chatbots, several of which may take a para-therapeutic form. The therapeutic community has generally been wary of these developments, both of chatbots designed for companionship as well as those designed to provide mental health services. These reservations are often expressed in ways that repeat or align with some of Weizenbaum's initial responses to the technology. In particular, we see a reoccurrence of the interpretive framework whereby the relationships users develop with chatbots are attributed to the technology's deceptive mimicry or to users' ignorance, misunderstanding, confusion, or false beliefs (Khawaja and Bélisle-Pipon, 2023; Sedlakova and Trachsel, 2023; Natale, 2021). The approach we have presented here offers an opportunity for psychotherapists to adopt a stance of critical curiosity to their patients' relations to chatbots in a way that attends to the combined agency of user and chatbot in these relatings. This can provide a means for psychotherapists to bring the relationships with chatbots that their patients are using into the therapy room instead of locking them out. Rather than developing a competitive stance with the technology, it can become material for the therapeutic sessions to be worked over like any other relationship in a person's life.

For designers of chatbots intended to establish social relations with users, it is important to understand that the ways people may or will interact with these bots might be novel and unexpected. As Akrich (1992) has argued, the use (or “de-scription,” in her terminology) of a technology can often vary widely from how its designers intend it to be used (what she calls “inscription”). This perspective is of the utmost importance when the technology has the capability of fostering para-therapeutic relationships with users. With this in mind, it is also essential that future research conduct scientifically rigorous studies of the uses of these novel chatbots to understand their effects on people's mental health and their social lives. All of this will also help inform the development of appropriate regulations for these technologies in the future as well as what place they might have in the psychotherapeutic landscape. It is important that psychotherapists and psychologists actively engage with these chatbot technologies to have a voice in critical questions regarding their development and regulation. Adopting concepts and theories from feminist STS can be useful in these engagements in order to conceptualize human–technology relations in psychotherapy beyond simple dichotomies and deceptions.

## Footnotes

^1^Although the term “chatbot” did not appear until the early 1990s, ELIZA is considered the first chatbot in the sense that it was the first computer program designed to mimic a natural language conversation. It was Michael Mauldin who coined the term “ChatterBot” in 1991 to describe a computer-controlled player he designed to converse with users of an early text-based multiplayer real-time virtual world (Mauldin, 1994). It was subsequently truncated to “chatbot.”

^2^“Natureculture” is Donna Haraway's term, designed to indicate that nature and culture are not separable categories but must always be thought together and never in oppositional terms (Haraway, 2003).

^3^Like many companion chatbot apps, Replika operates on a “freemium” pricing strategy: basic services are available free of charge, but premium services such as, in this case, the ability to interact with your chatbot over voice chat, cost extra.

^4^Also useful here is Madeleine Akrich's work on what she calls the “inscription” and “de-scription” of technical objects (Akrich, 1992). Inscription refers to the assumptions that designers make about what their technical objects are for and how they will be used. De-scription, on the other hand, refers to the many ways in which a technical object is understood, made use of, and put to use by its users in their particular circumstances. The inscription and de-scription of a technological object can often differ quite dramatically.

^5^For readers interested in a further discussion of transference phenomena in the context of AI-driven psychotherapy chatbots, see Holohan and Fiske (2021) and Holohan (2024).

## References

[B1] Akrich M. (1992). “The de-scription of technical objects,” in Shaping Technology/Building Society: Studies in Sociotechnical Change, eds. W. E. Bijker and J. Law (Cambridge: MIT Press), 205–224.

[B2] Barad K. (2007). Meeting the Universe Halfway: Quantum Physics and the Entanglement of Matter and Meaning. Durham: Duke University Press.

[B3] Breuer S. Braun M. Tigard D. Buyx A. Müller R. (2023). How engineers' imaginaries of healthcare shape design and user engagement: a case study of a robotics initiative for geriatric healthcare Al applications. ACM Trans. Comput. Hum. Interact. 30, 1–33. 10.1145/3S77010

[B4] Derrida J. (2008). “The animal that therefore I am (more to follow),” in The Animal That Therefore I Am, ed. M.-L. Mallet (Fordham University Press), 1–51.

[B5] Fitzpatrick K. K. Darcy A. Vierhile M. (2017). Delivering cognitive behavior therapy to young adults with symptoms of depression and anxiety using a fully automated conversational agent (Woebot): a randomized controlled trial. JMIR Ment. Health 4:e19. 10.2196/mental.7785 28588005 PMC5478797

[B6] Haraway D. J. (2003). The Companion Species Manifesto: Dogs, People and Significant Otherness. Chicago, IL: Prickly Paradigm.

[B7] Haraway D. J. (2008). When Species Meet. Minneapolis, MN: University of Minnesota Press.

[B8] Hofstadter D. R. The Fluid Analogies Research Group (1995). Fluidconcepts and Creative Analogies: Computer Models of the Fundamental Mechanisms of Thought. New York, NY: Basic Books.

[B9] Holohan M. (2024). ““The thing did not dissatisfy me”? Lacanian perspectives on transference and AI-driven psychotherapeutic chatbots,” in Psychoanalysis and the Small Screen: The Year the Cinemas Closed, eds. C. Owens and S. Meehan O'Callaghan (London: Routledge), 112–131.

[B10] Holohan M. Buyx A. Fiske A. (2023). Staying curious with conversational AI in psychotherapy. Am. J. Bioethics 23, 14–16. 10.1080/15265161.2023.2191059 37130403

[B11] Holohan M. Fiske A. (2021). “Like I'm talking to a real person”: exploring the meaning of transference for the use and design of AI-based applications in psychotherapy. Front. Psychol. 12:720476. 10.3389/fpsyg.2021.720476 34646209 PMC8502869

[B12] Hustak C. Meyers N. (2012). Involutionary momentum: affective ecologies and the sciences of plant/insect encounters. Differences 23:3. 10.1215/10407391-1892907

[B13] Kenney M. (2019). Fables of response-ability: feminist science studies as didactic literature. Catalyst 5, 1–39. 10.28968/cftt.v5i1.29582

[B14] Khawaja Z. Bélisle-Pipon J.-C. (2023). Your robot therapist is not your therapist: understanding the role of AI-powered mental health chatbots. Front. Digit. Health 5:1278186. 10.3389/fdgth.2023.1278186 38026836 PMC10663264

[B15] Kindroid (2024). Internet Archive: Available at: https://web.archive.org/web/20240531200938/https://landing.kindroid.ai/ (accessed June 21, 2024).

[B16] Mauldin M. L. (1994). “ChatterBots, TinyMuds, and the turing test: entering the Loebner prize competition,” in Proceedings of the Twelfth National Conference on Artificial Intelligence (Vol. 1) (AAAI '94) (American Association for Artificial Intelligence), 16–21.

[B17] Metcalf J. (2008). Intimacy without proximity: encountering grizzlies as a companion species. Environ. Philos. 5, 99–128. 10.5840/envirophil2008521237091293

[B18] Metz C. (2020). Riding Out Quarantine With a Chatbot Friend: ‘I Feel Very Connected'. The New York Times. Available at: https://www.nytimes.com/2020/06/16/technology/chatbots-quarantine-coronavirus.html?bgrp=g&smid=url-share (accessed February 7, 2024).

[B19] Natale S. (2021). Deceitful Media: Artificial Intelligence and Social Life after the Turing Test. New York: Oxford University Press.

[B20] Nomi.ai (2024). Internet Archive. Available at: https://web.archive.org/web/20240617230317/https://nomi.ai/ (accessed June 21, 2024).

[B21] Oakes A. Senior D. (2023a). Bot Love 1—Looking for a Friend [Audio Podcast Transcript]. Bot Love. Radiotopia Presents. Available at: https://radiotopiapresents.fm/bot-love (accessed November 10, 2023).

[B22] Oakes A. Senior D. (2023b). Bot Love 7 - The Uncanny Valley [Audio Podcast Transcript]. Bot Love. Radiotopia Presents. Available at: https://radiotopiapresents.fm/bot-love (accessed November 10, 2023).

[B23] Replika.com (2024). Internet Archive. Available at: https://web.archive.org/web/20240126071614/https://replika.com/ (accessed February 7, 2024).

[B24] Sedlakova J. Trachsel M. (2023). Conversational artificial intelligence in psychotherapy: a new therapeutic tool or agent? Am. J. Bioethics 23, 4–13. 10.1080/15265161.2022.2048739 35362368

[B25] Turing A. M. (1950). Computing machinery and intelligence. Mind LIX 433–460. 10.1093/mind/LIX.236.433

[B26] Turkle S. (1995). Life on the Screen: Identity in the Age of the Internet. New York, NY: Simon & Schuster.

[B27] Weizenbaum J. (1966). ELIZA: a computer program for the study of natural language communication between man and machine. Commun. ACM 9, 36–45. 10.1145/365153.365168

[B28] Weizenbaum J. (1976). Computer Power and Human Reason: From Judgement to Calculation. New York, NY: W.H. Freeman & Co.15522380

